# Effects of electroacupuncture on the incidence of postoperative supraventricular arrhythmia and sleep quality in patients undergoing thoracoscopic surgery: a randomized controlled trial

**DOI:** 10.3389/fneur.2025.1580759

**Published:** 2025-11-20

**Authors:** Jie Liu, Longfei Ding, Wuhua Ma, Jiyong Wang, Xiuling Song, Ying Cao, Caineng Wu

**Affiliations:** 1 First Clinical Medical College, Guangzhou University of Chinese Medicine, Guangzhou, China; 2 Department of Anesthesiology, The First Affiliated Hospital of Guangzhou University of Chinese Medicine, Guangzhou, China; 3 Guangdong Clinical Research Academy of Chinese Medicine, Guangzhou, China; 4 Department of Thoracic Surgery, The First Affiliated Hospital of Guangzhou University of Chinese Medicine, Guangzhou, China; 5 NMPA Key Laboratory for Research and Evaluation of Drug Metabolism, Guangdong Provincial Key Laboratory of New Drug Screening, School of Pharmaceutical Sciences, Southern Medical University, Guangzhou, China

**Keywords:** electroacupuncture, thoracoscopic surgery, supraventricular tachycardia, sleep disturbance, lung cancer

## Abstract

**Background:**

Supraventricular arrhythmia and sleep disturbance frequently occur after thoracoscopic surgery for lung cancer. The present study is designed to evaluate the hypothesis that electroacupuncture is an effective treatment of supraventricular arrhythmia and sleep disorders following thoracoscopic lung cancer surgery.

**Methods:**

Adult patients scheduled for single-port thoracoscopic lung cancer surgery were randomly assigned to the Electroacupuncture (EA) and control groups. The primary outcome of this trial was the incidence of new-onset supraventricular tachycardia (SVT) including atrial flutter, atrial fibrillation, atrial tachycardia and atrioventricular junctional tachycardia during the first 24 h after surgery.

**Results:**

The authors analyzed 77 patients (EA, 38; control, 39). The incidence of new-onset SVT was significantly lower in the EA group compared with the control group during the first 24 postoperative hours; 4 (10.5%) vs. 13 (33.3%), respectively, *p =* 0.02. Patients in the EA group had longer total sleep time (119.0 vs. 209.5, *p =* 0.02), longer duration of nonrapid eye movement sleep on the first postoperative night (*p* < 0.05). The awake time was significantly shorter compared with the control group (134.5.0 vs. 225.0, *p =* 0.01). Dosage of remifentanil and incidence of intraoperative hypotension were significantly reduced in the EA group (911.1 vs. 1095.9, *p =* 0.01). However, VAS scores after surgery did not differ between groups. In all the patients recruited, adverse effects such as redness, swelling and inflammatory reactions were not observed at the acupuncture site.

**Conclusion:**

The results of this study suggest that perioperative electroacupuncture treatment could be a promising strategy to reduce the incidence of new-onset SVT and improve sleep disturbance in patients undergoing thoracoscopic surgery for lung cancer. This potential impact on future treatments should inspire hope and optimism in the medical community.

**Clinical trial registration:**

https://www.chictr.org.cn/indexEN.html, identifier ChiCTR2300077984.

## 1 Introduction

As a malignant tumor, lung cancer has a high incidence and fast growth rate not only in China but also all over the world. Thoracoscopic surgery has been widely adopted for lung cancer. Despite its small incision and minimal tissue trauma, the incidence of postoperative arrhythmias and sleep disturbances remains high (1, 2). Our previous study showed that new-onset arrhythmia was observed in about 40% of the patients undergoing thoracoscopic surgery, the most common of which was postoperative supraventricular tachycardia (POSVT) (31.8%) (3). This may increase the risk of hemodynamic instability and endanger the postoperative safety of lung cancer patients (4). Sleep disturbance is also a common symptom in patients with lung cancer, which may contribute to a decelerated postoperative recovery (5).

Electroacupuncture (EA), a nonpharmacological adjunctive intervention, has been employed in clinical practice and has proved to be an effective treatment for patients with atrial fibrillation (6–8). Moreover, although some studies have reported that the application of EA is effective in improving sleep quality and regulating circadian sleep rhythms (9), whether the use of EA is associated with a lower incidence of postoperative arrhythmias and better sleep quality in patients after thoracic surgery has not yet been confirmed. To verify, we performed this randomized clinical trial to explore the efficacy of EA in preventing supraventricular arrhythmia and sleep disorders undergoing thoracic surgery.

## 2 Methods

### 2.1 Ethics approval and informed consent

The present study was approved by the Ethics Committee of First Affiliated Hospital of Guangzhou University of Chinese Medicine (Approval No. K-2023-128) in November 2023. It was registered at the Chinese Clinical Trial Registry (Registration No. ChiCTR2300077984). Written informed consent was obtained from each patient enrolled. The work has been reported per the CONSORT criteria.

### 2.2 Participants

Inclusion criteria were: ASA (American Society of Anesthesiologists) physical status 1 to 3, single-port thoracoscopic lung cancer surgery; age between 20 and 80 years old; volunteered to participate in this trial. Exclusion criteria included any cardiac, pulmonary, hepatic or renal dysfunction; pre-existed cardiac arrhythmia; history of thoracic surgery or sleep disturbances (subjective sleep quality in the participant’s daily life was assessed according to the preoperative Sleep Quality Numerical Rating Scale, which is a numerical scale from 0 to 10, with 0 indicating excellent sleep quality and 10 indicating complete sleep deprivation throughout the night. A score of 6 or higher on the Sleep Quality Numerical Rating Scale indicates a sleep disorder, while a score of less than 6 indicates a non-sleep disorder. Sleep disorder was excluded based on this criterion) (10), perioperative electrolyte disturbances; abnormal thyroid function or mental impairment; usage of antiarrhythmic drugs in 3 days before the surgery; skin infection or nerve damage at the selected acupuncture points; patients already enrolled in other clinical trials.

### 2.3 Randomization and blinding

A statistical researcher generated Randomization sequences using SPSS in a 1:1 ratio. According to the randomization sequence, eligible patients were randomly divided into one of the two groups (the EA or the control group). Group allocation was concealed in serially numbered, sealed opaque envelopes by a researcher blind to the groups. On the operation day, the EA provider opened the envelope and documented the predetermined group allocation of each patient in a secluded place and a consistent tone. The surgeons, anesthesiologists, patients, data collectors and statisticians were unaware of the groups throughout the study.

### 2.4 Interventions

The selection of EA acupoints is determined in consultation with an acupuncturist expert. The location of Neiguan (PC6) and Gongsun (SP4) are described in The National Standards for Acupoint Location (11). In the EA group, patients initially received EA treatment 30 min before anesthetic induction at Neiguan (PC6) and Gongsun (SP4) acupoints on the surgical side of the patient. This treatment lasted until the end of the surgery and was repeated for 30 min at the 24th hour after the surgery. Sterilized and disposable needles (size 0.25 × 25 mm, Suzhou Medical Supplies Factory Co., Ltd., China) were inserted to a depth of 3–5 mm at Neiguan (PC6) and Gongsun (SP4) acupoints on the surgical side of the patient. The needles were carefully manipulated until a de qi sensation (most commonly fullness, numbness, and soreness) was experienced by the patient. Then, the needles were connected to an EA stimulator (SDZ-II, Suzhou Medical Supplies Factory Co., Ltd., China). The stimulation was performed with a frequency of 2/100 Hz and at the maximum current the patient can tolerate, usually between 8 and 12 mA. In the control group, needles were inserted to the same depth at non-specific acupoints near PC6 and SP4. The acupuncturist then used the same operation as PC6 and SP4 in the EA group.

After sterilizing the skin in the surgical area, the areas were covered with sterile sheets to ensure blinding of the study. All the EA treatment and anesthesia induction in both groups were performed by the same qualified anesthesiologist with the patients placed in a supine position. The anesthesiologist was specially trained in acupuncture and had more than 5 years of experience in acupuncture. Another anesthesiologist blind to the grouping was responsible for the subsequent anesthesia management. All the surgeries were performed by the same surgical team. The surgeons, anesthesiologist, patients, data collectors, ECG physicians, sleep physicians and statisticians were unaware of the groups throughout the study. Group assignments were not revealed to outcome assessors, and they did not participate in the EA treatment.

### 2.5 Anesthetic technique

On the day of the surgery, noninvasive blood pressure, electrocardiography and pulse oximetry were routinely monitored after patient admission to the operating room. After preoxygenation for 5 min, intravenous induction was started with 0.3 μg kg^−1^ sufentanil, 1 to 2.5 mg kg^−1^ propofol and 0.1 mg kg^−1^ vecuronium bromide. Patients were then intubated with a double-lumen tube of appropriate size for one-lung ventilation. Auscultation and fiberoptic bronchoscopy were performed to confirm the correct placement of the tube. The ventilator parameters were: oxygen flow rate 2 L min^−1^, FiO2 60%, VT 6 to 8 mL kg^−1^, RR 12 times min^−1^, to maintain a normal end-tidal carbon dioxide pressure. Remifentanil 0.1–0.2 μg kg^−1^ min^−1^and sevoflurane 1–3% were used in combination for anesthesia maintenance. The infusion rate of remifentanil and sevoflurane was adjusted to keep bispectral index values between 40 and 60. The neuromuscular blockade was maintained by the addition of vecuronium intermittently. Before surgical skin incision, parecoxib sodium 50 mg was injected intravenously. At 5 min before the end of the surgery, 0.1 μg kg^−1^ sufentanil was administered for analgesia transition and 0.5% (0.2 mL kg^−1^) ropivacaine was infiltrated into the surgical wound. A patient-controlled intravenous analgesia (PCIA) pump was connected for postoperative analgesia (sufentanil 1.5 μg/mL, PCIA dose: 3 mL, background dose: 2 mL/h, interval: 20 min, and duration: 2 days).

Intraoperative hypotension, defined as more than 20% reduction in systolic blood pressure from the baseline (measured before anesthesia induction) was treated by adequate infusion and intravenous ephedrine or noradrenaline. Bradycardia (heart rate less than 45 bpm), was treated with 0.5 mg atropine intravenously during the procedure. In addition, hypertension or tachycardia (more than 20% increase from baseline) was managed by increasing the concentration of sevoflurane or the rate of remifentanil infusion and intravenous injection of urapidil.

### 2.6 Postoperative period

Patients were closely monitored in the thoracic surgery ward after surgery. The ECG with a multichannel 24-h Holter ECG was continuously monitored during the first 24 h postoperatively. An experienced physician, who was unaware of the group allocation, was responsible for analyzing the 24-h Holter results with a DMS ECG Holter System (DM Systems Co, Ltd., Beijing, China). SVT including atrial flutter, atrial fibrillation and other SVT was documented. β-receptor blockers were used to terminate serious SVT by a thoracic physician blind to the grouping.

During the first postoperative night, sleep status was monitored using Lifelines Trakit TM Sleep (an ambulatory sleep recorder, 7 Clarendon Court, Over Wallop, Nr. Stockbridge, Hants, UK). An experienced physician who was unaware of the grouping analyzed the sleep condition. Sleep pattern was divided into rapid eye movement (REM) sleep and non-REM sleep (NREM). NREM sleep was further divided into stage N1, stage N2 and stage N3 sleep.

### 2.7 Study outcomes

The primary outcome of this trial was the incidence of SVT including atrial flutter, atrial fibrillation, atrial tachycardia and atrioventricular junctional tachycardia during the first 24 h after surgery. Supraventricular ectopic beat and ventricular premature beat were also documented. Total sleep time (TST), defined as the sum of time spent in sleep during the first postoperative night (from 18:00 to 08:00), time of REM sleep and time of non-REM sleep were recorded as the secondary outcomes. Awake time was the total time spent awake between the initial sleep onset and the last sleep end during the first postoperative night. By dividing TST by the sum of TST and awake time sleep efficiency was calculated. Meanwhile, usage of β-receptor blockers and postoperative pain score were recorded. Postoperative pain scores were expressed by visual analog scores (VAS).

### 2.8 Sample size

The primary outcome of this study was the incidence of POSVT during the first 24 h after thoracoscopic surgery. Our previous study had shown that the incidence of POSVT was 40% in patients undergoing non-cardiac thoracic surgery (1). Due to scarce similar studies, we carried out a pilot study before the start of this trial. We found that the incidence of POSVT after thoracoscopic surgery was reduced to about 11% with EA treatment. Therefore, it was supposed that POSVT would occur in 40% of the control group and 11% of the EA group. With a two-sided significance level of 5%, power of 80% and drop-out rate of 10%, the minimum number of patients required to detect a significant difference between the two groups was set at 80 (40 patients in each group).

### 2.9 Statistical methods

Continuous variables were presented as mean (SD) for normal distribution or median (IQR (interquartile range) [range]) for skewed distributions. The normality of data was previously tested with Shapiro–Wilk test. Categorical variables were reported as counts (percentage). Continuously normally distributed data were compared using independent samples *t*-test and continuously nonnormally distributed data using Mann–Whitney *U*-test. Categorical variables were analyzed using χ^2^ test or Fisher’s exact test. Analyses were performed using SPSS (IBM SPSS Statistics Version 22; SPSS Inc., Chicago, IL, United States). Results were considered statistically significant with *p* values less than 0.05.

## 3 Results

**Figure 1:**
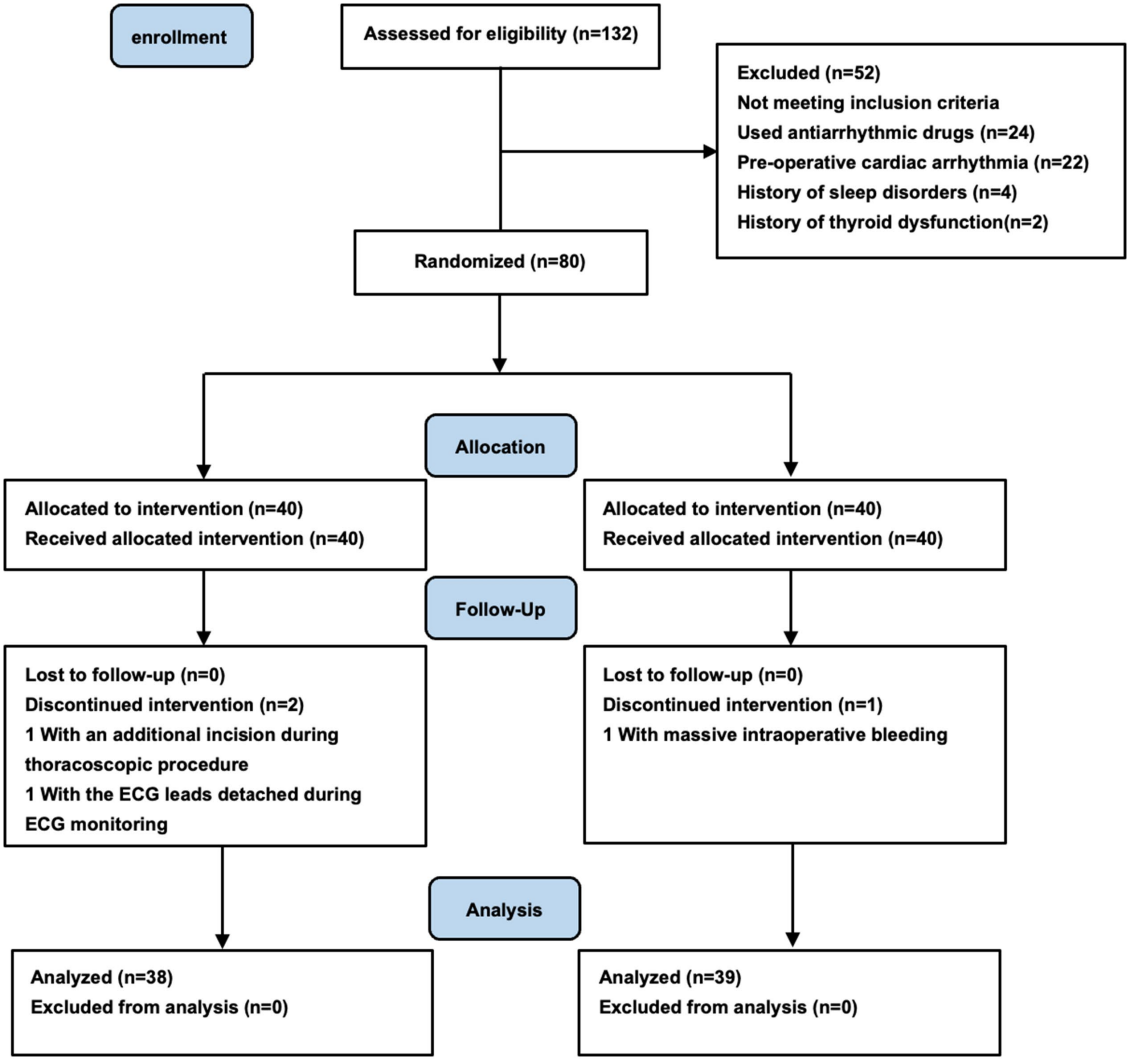
Study flow diagram.

Between December 2023 and July 2024, 132 patients were screened for eligibility. 52 (39%) were excluded according to the exclusion criteria, and 80 patients were enrolled in this study. In the EA group, one patient had an additional incision during thoracoscopic procedure, and another patient had the ECG leads detached during ECG monitoring. Therefore, these two patients were withdrawn from the study. In the control group, there was also a patient who dropped out of the study due to massive intraoperative bleeding and the requirement for ICU delivery postoperatively (Figure 1). Finally, 77 patients finished the study and were included in the analysis. In all the patients recruited, adverse effects such as redness, swelling and inflammatory reactions were not observed at the acupuncture site.

There were no significant differences between groups in baseline demographics and patient characteristics (Table 1). The dose of remifentanil was significantly reduced in the EA group; 1095.9 (362.2) vs. 911.1 (233.8) μg, *p =* 0.01. The incidence of intraoperative hypotension in the EA group was significantly lower than in the control group; 15 (38.5%) vs. 6 (15.8%), *p =* 0.05. No other significant differences were observed between the groups during anesthesia (Table 2).

**Table 1:** Baseline demographics and characteristics.

Characteristic	Control group, *n* = 39	EA group, *n* = 38	Difference in mean, medians or proportions (95%CI)	*P* value
Age (year)	61.3 ± 10.5	60.3 ± 11.9	1.02 (−4.06 to 6.01)	0.69
Sex (M/F)	20/19	15/23	0.12 (−0.10 to 0.34)	0.41
Weight (kg)	60.2 ± 10.0	60.4 ± 6.0	−0.16 (−3.91 to 3.58)	0.93
Height(cm)	161.4 ± 7.3	161.7 ± 7.7	−0.22(−3.64 to 3.19)	0.90
ASA, *n*				
I	13 (33.3%)	8 (21.1%)	0.12 (−0.07 to 0.32)	0.23
II	22 (56.4%)	29 (76.3%)	−0.20 (−0.41 to 0.01)	0.07
III	4 (10.3%)	1 (2.6)	0.08 (−0.03 to 0.18)	0.18
History of smoking, *n*	14 (35.9%)	11 (28.9%)	0.07 (−0.14 to 0.28)	0.52
Type of surgery, *n*				
Segmentectomy	25 (64.1%)	22 (57.9%)	0.06 (−0.16 to 0.28)	0.58
Lobectomy	14 (35.9%)	16 (42.1%)	−0.06 (−0.28 to 0.16)	0.58
SQ-NRS	2 [1 to 3]	2 [1 to 3]	0 (0 to 0)	0.74

Values are mean ± SD or number (proportion) or median [IQR]. EA, electroacupuncture; M/F, Male/Female; SQ-NRS, Sleep Quality Numerical Rating Scale; ASA, American Society of Anaesthesiologists; IQR, interquartile range; CI, Confidence Interval.

**Table 2:** Intraoperative data.

Intraoperative data	Control group (*n* = 39)	EA group (*n* = 38)	Difference in mean, medians or proportions (95%CI)	*p* value
Duration of surgery (min)	153 ± 53	146 ± 46	6.87 (−15.54 to 29.28)	0.54
Duration of OLV (min)	137 ± 53	130 ± 45	6.27 (−16.13 to 28.67)	0.58
Dose of propofol (mg)	116 ± 24	119 ± 14	−2.54 (−11.32 to 6.25)	0.57
Dose of vecuronium (mg)	8 [7 to 8]	8 [8 to 9]	0.00 (−1.00 to 0.00)	0.07
Dose of sufentanil (μg)	24.1 ± 4.0	24.0 ± 2.8	0.02 (−1.56 to 1.61)	0.98
Dose of remifentanil (μg)	1095.9 ± 362.2	911.1 ± 233.8	184.82 (46.02 to 323.62)	0.01
Blood loss (ml)	25 [20 to 30]	28 [20 to 36]	−2.50 (−5.00 to 0.00)	0.33
Intraoperative infusion volume (ml)	1,450 [1,200 to 1,600]	1,350 [1,062 to 1,500]	100 (−50 to 250)	0.14
Intraoperative hypertension	2 (5.1%)	0 (0%)	0.05 (−0.02 to 0.12)	0.25
Intraoperative hypotension	15 (38.5%)	6 (15.8%)	0.23 (0.04 to 0.42)	0.05
Intraoperative tachycardia	2 (5.1%)	0 (0%)	0.05 (−0.02 to 0.12)	0.25
Intraoperative bradycardia	4 (10.3%)	1 (2.6%)	0.08 (−0.03 to 0.18)	0.37

Values are mean ± SD, number (proportion) or median [IQR]. EA, electroacupuncture; OLV, one lung ventilation; IQR, interquartile range.

Postoperative arrhythmia was commonly observed in patients after thoracoscopic surgery for lung cancer. Compared with the EA group, the incidence of new-onset SVT was significantly higher in the control group during the first 24 h; 13 (33.3%) vs. 4 (10.5%), *p =* 0.02 (Table 3). There were no significant differences in the incidence of supraventricular ectopic beat and ventricular premature beat between groups (Table 3). A significantly more frequent use of β-blockers during the first 24 postoperative hours was observed in the control group; 11 (28.2%) vs. 3 (7.9%), *p =* 0.04 (Table 3).

**Table 3:** Incidence of postoperative arrhythmia.

	Control group, (*n* = 39)	EA group, (*n* = 38)	Difference proportions (95%CI)	*P* value
POSVT	13 (33.3%)	4 (10.5%)	0.18 (0.01 to 0.35)	0.02
Segmentectomy	8 (32.0%)	2 (9.1%)	0.23 (0.01 to 0.45)	0.12
Lobectomy	5 (35.7%)	2 (12.5%)	0.23 (−0.07 to 0.53)	0.29
SVEBs	23 (59.0%)	18 (47.4%)	0.12 (−0.11 to 0.34)	0.31
VPBs	26 (66.7%)	17 (44.7%)	0.22 (0.00 to 0.44)	0.05
Use of β-blockers	11 (28.2%)	3 (7.9%)	0.20 (0.04 to 0.37)	0.04

Values are number (proportion). EA, electroacupuncture; POSVT, postoperative supraventricular tachycardia; SVEB, supraventricular ectopic beat; VPB, ventricular premature beat; CI, Confidence Interval.

There were significant differences in the TST and sleep efficiency during the first postoperative night between the two groups; 119 (48–193 [10–294]) vs. 209.5 (73.8–261.5 [16–349]), *p =* 0.02 and 0.34 (0.14–0.57 [0.03–0.95]) vs. 0.60 (0.23–0.79 [0.03–0.95]), *p =* 0.02, respectively. Compared with the EA group, patients in the control group experienced significantly longer awake time; 225.0 (153.0–316.0 [17–372]) vs. 134.5 (72.8–248.3 [16–349], *p =* 0.01). The sleep duration of stage N1 and stage N2 was significantly longer in the EA group; 95.0 (38.0–123.0 [10–208]) vs. 135 (56.2–177.3 [9–242]), *p =* 0.05 and 28 (12–58 [0–125]) vs. 68.5 (21.0–94.3 [0–139]), *p =* 0.02, respectively (Table 4).

**Table 4:** Sleep stages.

	Control group, (*n* = 39)	EA group, (*n* = 38)	Difference in medians (95%CI)	*P* value
TST (min)	119 [48 to 193]	209.5 [73.8 to 261.5]	−90.5 (−109 to −10)	0.02
Sleep efficiency	0.34 [0.14 to 0.57]	0.60 [0.23 to 0.79]	−0.26 (−0.32 to −0.02)	0.02
Awake time (min)	225.0 [153.0 to 316.0]	134.5 [72.8 to 248.3]	90.5 (14 to 112)	0.01
Stage N1 (min)	95.0 [38.0 to 123.0]	135 [56.2 to 177.3]	−40 (−59 to −1)	0.05
Stage N2 (min)	28 [12 to 58]	68.5 [21.0 to 94.3]	−40.5 (−50 to −5)	0.02
Stage N3 (min)	0 [0 to 0]	0 [0 to 2]	0 (0 to 0)	0.08
REM (min)	0 [0 to 0]	0 [0 to 0]	0 (0 to 0)	>0.99

Values are median IQR [range].

Values are median [IQR]. EA, electroacupuncture; TST, total sleep time; REM, rapid eye movement sleep; CI, Confidence Interval.

**Figure 2:**
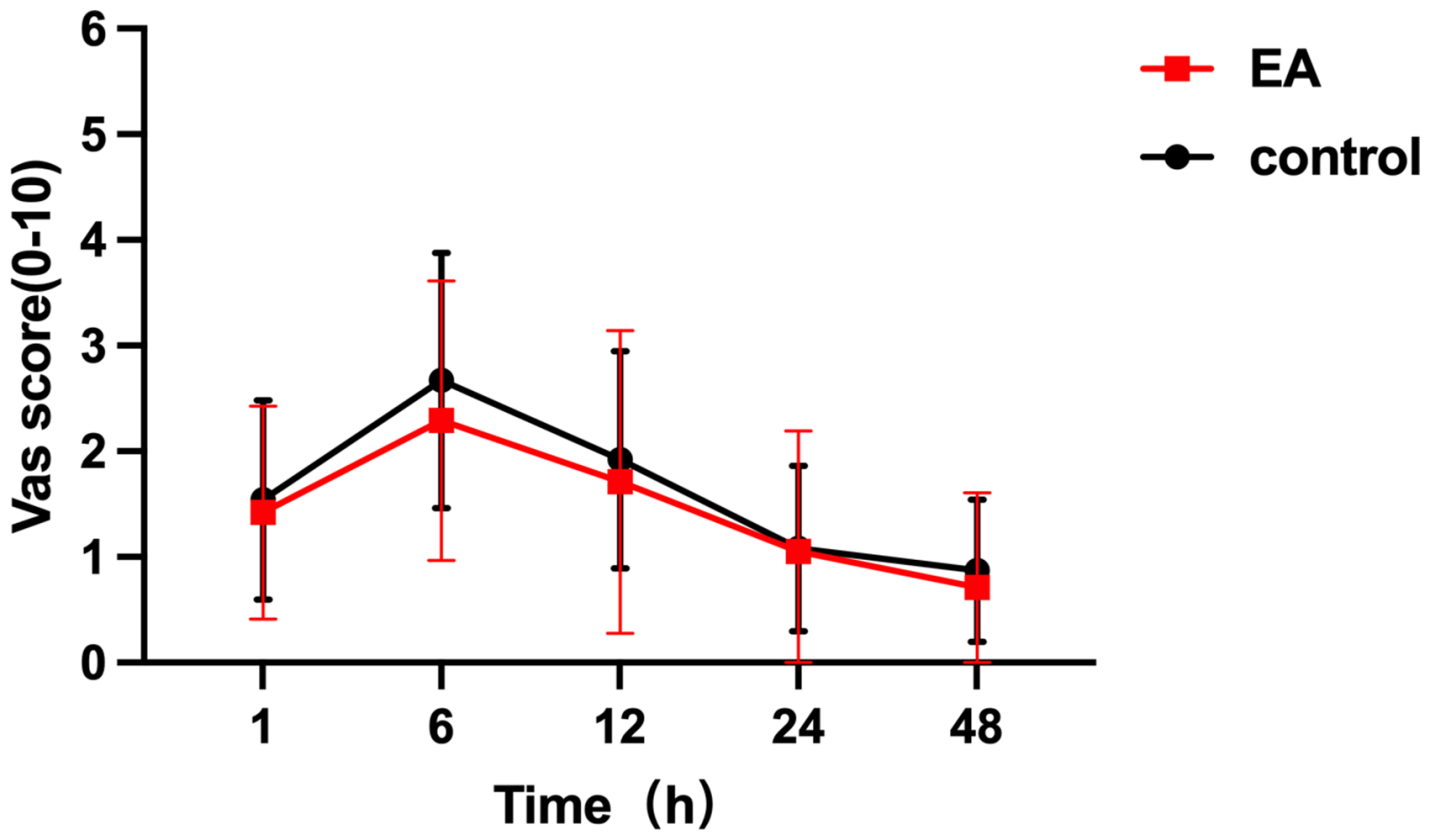
VAS scores from 1 to 48 h postoperatively in the EA group and the control group. No significant difference between groups.

From 1 h to 48 h after surgery, the VAS scores were all comparable between the two groups and postoperative pain was well controlled (Figure 2). Comparisons at different time points showed that in the control group, the VAS scores at 1 h postoperatively differed significantly from those at 6 h, 12 h, and 48 h postoperatively, with mean differences and 95% CI of −1.38 (−1.79 to −0.98), *p* < 0.001; −0.64 (−1.05 to −0.23), *p* < 0.001; 0.41 (0.00–0.82), *p =* 0.05. In the EA group, significant differences of VAS scores were found only at 6 h and 48 h postoperatively, with results of −0.87 (−1.40 to −0.34), *p* < 0.001; 0.71 (0.28–1.14), *p* < 0.001.

Univariate and multivariate logistic regression analyses examined the relationship between EA and POSVT (Table 5). In univariate analysis, EA was a protective factor for POSVT (OR 0.24, 95% CI 0.07–0.81, *p* = 0.02). Other important variables in the univariate analysis included age, duration of surgery, duration of OLV (one lung ventilation), dose of propofol, dose of sufentanil, dose of remifentanil, blood loss, ASA classification, types of surgery. After adjusting for age, duration of surgery, duration of OLV, doses of propofol, dose of sufentanil, dose of remifentanil, blood loss, ASA classification, and types of surgery, EA remained protective against POSVT (OR 0.18, 95% CI 0.04–0.77, *p* = 0.02). In contrast, in both univariate and multivariate logistic analyses, variables other than EA did not seem to significantly affect on POSVT.

**Table 5:** Univariate and multivariate analysis of factors associated with POSVT.

Variable	Univariate			Multivariate		
	OR	95% CI	*P*	OR	95% CI	*P*
Intervention						
Simulated EA		Reference			Reference	
EA	0.24	0.07 to 0.81	0.02	0.18	0.04 to 0.77	0.02
Age	1.05	0.99 to 1.11	0.10	1.03	0.96 to 1.11	0.44
Duration of surgery	1.00	1.00 to 1.02	0.23	1.02	0.90 to 1.15	0.80
Duration of OLV	1.00	1.00 to 1.02	0.22	1.02	0.90 to 1.15	0.79
Dose of propofol	1.00	0.97 to 1.02	0.77	1.00	0.95 to 1.06	0.84
Dose of sufentanil	1.02	0.88 to 1.19	0.79	0.99	0.72 to 1.35	0.93
Dose of remifentanil	1.00	0.99 to 1.00	0.37	1.00	0.99 to 1.00	0.28
Blood loss	0.99	0.95 to 1.03	0.64	0.94	0.87 to 1.01	0.91
ASA						
I	Reference			Reference		
II	1.85	0.46 to 7.36	0.39	4.44	0.64 to 30.62	0.13
III	4	0.46 to 34.92	0.21	7.61	0.40 to 145.30	0.18
Types of surgery						
Segmentectomy	Reference			Reference		
Lobectomy	1.54	0.52 to 4.56	0.44	1.17	0.30 to 4.68	0.82

SVT, supraventricular tachycardia; CI, confidence interval; EA, electroacupuncture; ASA, American Society of Anaesthesiologists; OLV, One Lung Ventilation.

**Figure 3:**
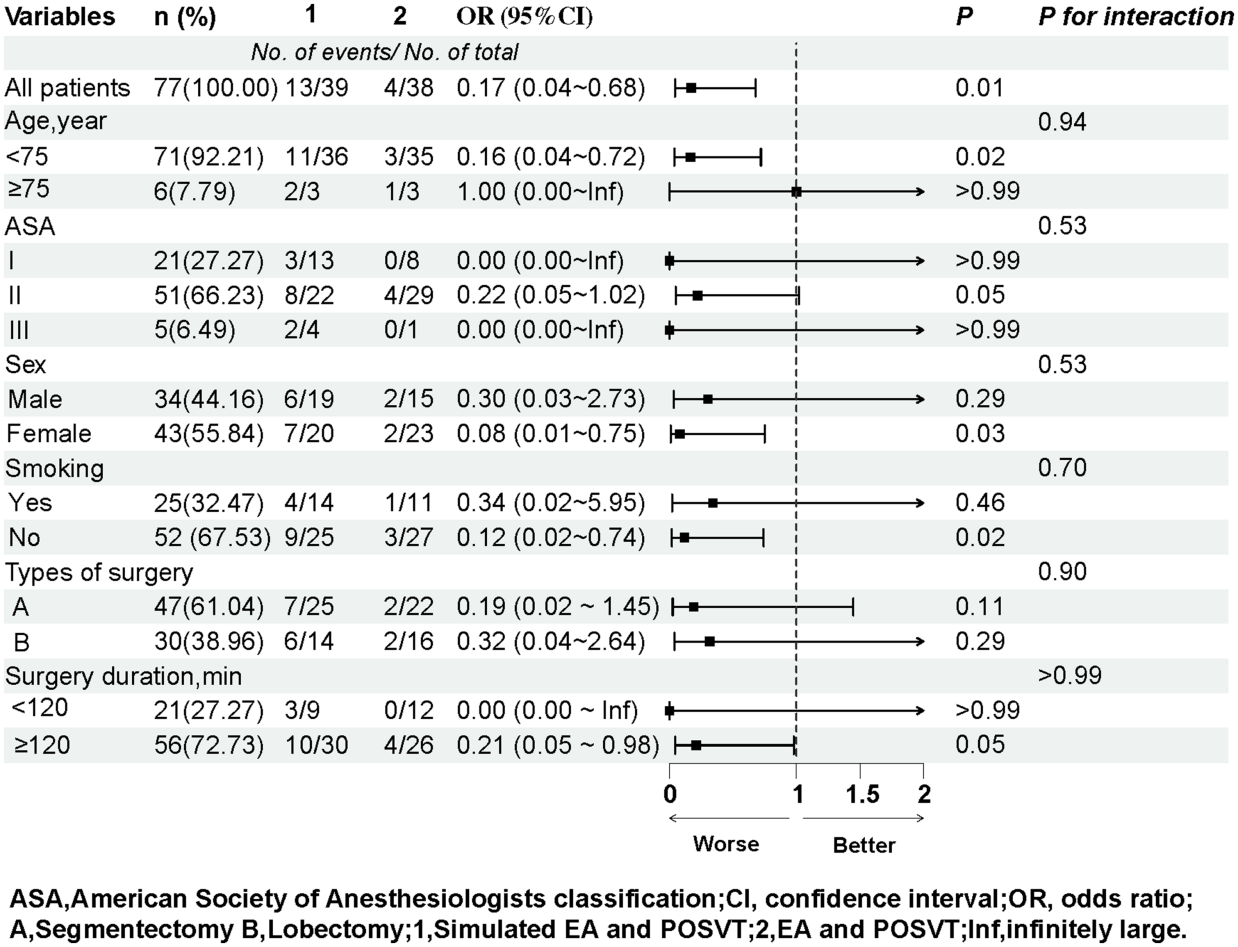
Subgroup analyses of the association between the EA and control group.

We performed subgroup analyses to explore the relationship between EA and POSVT in different subgroups (Figure 3). These subgroups were analyzed based on factors such as age, gender, ASA classification, smoking history, duration of surgery, and types of surgery to reveal significant associations between several factors and POSVT. However, these factors did not show significant associations with outcomes. No interactions were observed between subgroups. For detailed results of the subgroup analysis, please refer to Figure 3.

## 4 Discussion

In this study, we investigated the effect of EA treatment on the incidence of supraventricular arrhythmia and sleep disorders in patients undergoing thoracoscopic surgery for lung cancer. The main results indicated that peri-operative EA treatment was effectively reduced the incidence of POSVT and improved the sleep quality. It was also beneficial for maintaining intraoperative hemodynamic stability. Last not the least, the minimally invasive EA treatment appears to be safe and well tolerated in all the patients recruited. To the best of our knowledge, this is the first study to explore the effect of EA treatment on the prevention of SVT and sleep disorders in patients undergoing thoracoscopic surgery.

SVT has been recognized as a common complication after thoracoscopic surgery. It is associated with hemodynamic instability, prolonged hospital stay and increased risk of perioperative mortality (12, 13). Potential factors associated with POSVT included cardiac autonomic nervous system imbalance, oxidative activation, inadequate pain control and postoperative inflammatory response (14–17). Autonomic imbalance has been shown as the most frequent trigger of POSVT (18, 19). Our two previous studies showed that reducing in the tone of cardiac autonomic sympathetic nerves may help decrease the occurrence of POSVT (3, 20).

In 1958, traditional acupuncture was first used to complement the anesthetic. Acupuncture has been shown to enhance the rehabilitation of patients perioperatively, and it has also developed rapidly (21, 22).

In the present study, we found the beneficial effect of EA treatment in reducing SVT in patients after thoracoscopic surgery for lung cancer. The results were consistent with the study by Lomuscio et al. (23) Lomuscio et al. have proved that acupuncture helps decrease the recurrences of atrial fibrillation after electrical cardioversion and the antiarrhythmic efficacy of acupuncture was similar to that of amiodarone. In another study, the findings indicated that acupuncture may exert an antiarrhythmic effect in patients with both persistent and paroxysmal atrial fibrillation (24). The antiarrhythmic effect of acupuncture may be exerted by modulating the cardiac autonomic nervous system (25). Acupuncture has been widely proven effective in reducing the incidence of atrial fibrillation, both in basic research and clinical trials (25, 26). An experiment in a canine model suggested that acupuncture decreased cardiac sympathetic activity and suppressed atrial electrical remodeling by decreasing the levels of inflammatory cytokines in the atrium (27). In the present study, we chose Neiguan (PC6) and Gongsun (SP4) acupoints because they are related to the regulation of the cardiac autonomic nervous system in the theory of Traditional Chinese Medicine. While non-specific acupoints are not thought to have similar specific effects. Studies have noted that EA treatment may attenuate sympathoexcitatory cardiovascular responses by promoting the secretion of brain inhibitory neurotransmitters, including opioids and gamma-aminobutyric acid (28). Moreover, it has also been reported that EA treatment might affect the firing rate of the amygdala nucleus which exerts a modulatory function on the cardiac autonomic nervous system (29). Autonomic imbalance has been shown as the most common cause of POSVT (19). This may explain the mechanism by which EA treatment reduces the incidence of POSVT.

In addition, our results indicated that most patients experienced severe insomnia characterized by decreased TST and NREM sleep during the first postoperative night after thoracoscopic surgery for lung cancer. Postoperative sleep disorders are frequently observed in patients undergoing major surgery (30). Despite the high prevalence, insomnia was often not given enough attention and was not adequately treated due to the side effects of current medication treatment (31). Our present study demonstrated the significant therapeutic effects of EA on patients with postoperative insomnia. This is consistent with the results of another study on the effect of acupuncture on insomnia in breast cancer patients (32). The study showed that acupuncture could be considered as an effective management of chemotherapy-associated insomnia in breast cancer patients. Another study by Zhang *et al.* also investigated the clinical efficacy of acupuncture and found that acupuncture can significantly improve insomnia (33). The mechanisms of EA in treating insomnia are still under investigation. Clinical studies indicated that acupuncture may improve sleep quality through multiple pathways. Among them, autonomic nervous system plays a pivotal role in sleep physiology. Insomnia is usually accompanied by autonomic nervous dysfunction. Acupuncture may improve sleep quality by regulating the autonomic nervous system (34). Acupuncture can also regulate neurotransmitters. It may promote sleep quality by lowering cortisol levels in the stress response and increasing the level of 5-HT, a key inhibitory neurotransmitter involved in the regulation of the sleep cycle (35). Another mechanism of acupuncture in treating sleep disturbances is its anti-inflammatory effect. Studies have reported that EA treatment might alleviate the production of inflammatory cytokines, including IL-4 and IL-10 (36, 37). Inflammation is supposed to be associated with various sleep disorders (38). Therefore, EA treatment may promote sleep quality by reducing inflammatory cytokines.

Intraoperative hypotension is prevalent among patients undergoing thoracic surgery. We observed that the incidence of hypotension was as high as 38% during surgery, which may be due to the relatively advanced age of patients (more than 60 years old). We found that EA treatment can reduce the incidence of intraoperative hypotension. The hemodynamics of patients in the EA group seems to be more stable. This effect of EA treatment in patients undergoing thoracic surgery is rarely investigated and reported. The cardiac autonomic nervous system is suppressed during general anesthesia, and patients are predispositional to hypotension. EA may reduce the incidence of hypotension by regulation of an imbalanced autonomic nervous system (39). At present, the most common treatments for SVT after thoracoscopic surgery mainly include β-blockers, calcium channel blockers, and amiodarone. However, all of these medications are associated with hypotension and bradycardia, with occurrences as high as 49 and 25%, respectively (40, 41). They are usually contraindicated in patients with hemodynamic instability. Due to the hemodynamic advantages, EA may be a promising and feasible method to prevent POSVT during thoracic surgery.

Our data showed that EA treatment reduced intraoperative remifentanil consumption by almost 17%, indicating an analgesic effect of EA. Nevertheless, postoperative pain scores were similar in both groups. Although there is a study with similar results to ours exits (42), we cannot rule out the cause of heterogeneity. Several literatures have reported the analgesic effect of EA treatment and the mechanism may be related to endogenous opioid system activation (43–46).

In our study, the control group received EA treatment at non-acupoints as a reasonable placebo. Non-acupoints stimulation minimizes the differences in patient management between groups. It was reported that stimulation of acupoints could evoke antiarrhythmic and anti-inflammatory effect, while non-acupoint stimulation failed to exert the same effect (47).

### 4.1 Study limitations

Our study also has a few limitations. Firstly, this present study investigated the effect of EA treatment on the incidence of postoperative supraventricular arrhythmia and sleep quality in patients after thoracic surgery. However, the relationship between postoperative new-onset arrhythmias and sleep disturbances cannot be determined. Secondly, the sample size of this study is relatively small, and is a single-center clinical study. Our center is a tertiary hospital. The patients who come for surgery are relatively older and have more complex conditions and more comorbidities compared with the patients in lower-level hospitals. This may limit the generalizability of the results to other populations or settings and thus affect the study’s external validity. A larger multicenter clinical trial with a longer follow-up period to investigate the sustained effects of EA treatment on postoperative arrhythmias and sleep disturbances would be a valuable extension of this study.

## 5 Conclusion

In conclusion, POSVT and sleep disturbances are commonly observed in patients undergoing thoracoscopic surgery for lung cancer. EA treatment as a minimally invasive procedure appears to be effective in reducing the incidence of POSVT and improving postoperative sleep quality.

## Data Availability

The original contributions presented in the study are included in the article/supplementary material, further inquiries can be directed to the corresponding authors.
